# Identification and Characterization of Klebsiella pneumoniae from Farmed American Bullfrogs (*Rana catesbeiana*)

**DOI:** 10.1128/spectrum.03579-22

**Published:** 2023-01-05

**Authors:** Han Lin, Jie Ma, Jingyang Sun, Zhendong Qin, Biao Jiang, Wei Li, Qing Wang, Youlu Su, Li Lin, Chun Liu

**Affiliations:** a Guangzhou Key Laboratory of Aquatic Animal Diseases and Waterfowl Breeding, Innovative Institute of Animal Healthy Breeding, College of Animal Sciences and Technology, Zhongkai University of Agriculture and Engineering, Guangzhou, China; b Key Laboratory of Aquatic Animal Immune Technology of Guangdong Province and Key Laboratory of Fishery Drug Development of Ministry of Agriculture, Pearl River Fisheries Research Institute, Chinese Academy of Fishery Sciences, Guangzhou, China; University of California, Davis

**Keywords:** *Klebsiella pneumoniae*, American bullfrog, multidrug-resistant, multiple organ congestive enlargement

## Abstract

Klebsiella pneumoniae is a major cause of nosocomial infection and is considered a clinically important bacterium with antibiotic-resistant strains. There are few reports of K. pneumoniae infections in cultured aquatic animals, and no natural infection has been reported in amphibians. From September to October 2021, a high-mortality disease outbreak occurred in a pond-raised American bullfrog farm in Guangzhou, China. The infected bullfrogs were characterized by multiple organ congestive enlargement and inflammation. A pathogenic bacterium was isolated from the viscera of infected bullfrogs and confirmed to be K. pneumoniae by morphological, biochemical, and phylogenetic analyses. Infection experiments confirmed the virulence of the pathogenic strain against bullfrogs and tadpoles. A histopathological examination showed that the strain was harmful to multiple organs. Antibiotic resistance experiments indicated the isolate was a carbapenemase-producing multidrug-resistant K. pneumoniae (MDR-KP) strain. This study is the first report of K. pneumoniae infected American bullfrogs (Rana catesbeiana) and amphibians. These results will shed light on the pathogenicity of K. pneumoniae and help prevent and control K. pneumoniae infections in bullfrogs.

**IMPORTANCE** Klebsiella pneumoniae is recognized as the most common multidrug-resistant bacterial pathogen in humans, and little is known about its pathogenicity in aquatic animals. Recently, K. pneumoniae was found to cause substantial mortality and morbidity in American farm frogs. This was the first report of K. pneumoniae infecting amphibians. In this study, we analyzed the biochemical, growth, and phylogenetic characteristics of the K. pneumoniae strain and described the symptoms and pathological features of infected bullfrogs and tadpoles; this will provide useful data for the prevention and control of infectious diseases, which has been suggested to decrease economic losses in bullfrog farming and reduce the potential threat to public health posed by K. pneumoniae.

## INTRODUCTION

Klebsiella pneumoniae is a Gram-negative bacterium within the Enterobacteriaceae family, first described in 1882 (1). K. pneumoniae is the causative agent of many human infections, including neonatal sepsis, nosocomial pneumonia, liver abscess, and chronic intestinal diseases (2 to 4). In addition to its importance as a nosocomial pathogen, K. pneumoniae can be found in a wide range of hosts across various environmental niches (5). In terrestrial animals, this bacterium causes liver abscesses in nonhuman primates (6), subclinical mastitis in dairy cows (7), and respiratory infections in cats and dogs (8, 9). In aquatic animals, K. pneumoniae has been reported to cause pleuritis and suppurative bronchopneumonia in sea lions (10), liver and kidney necrosis, peritoneal hemorrhage in carp (11), and edematous bodies and hyperaemia in leeches (12). However, K. pneumoniae infection has rarely been reported in amphibians.

The American bullfrog (Rana catesbeiana) is a semiaquatic frog first described in 1802 (13). *R*. *catesbeiana* belongs to the order Anura in the class Amphibia and is one of 90 species in the genus *Rana* (14). American bullfrogs are native to North America, including the United States, Canada, and Mexico. In China, the species was introduced in the 1950s, and its aquaculture was gradually promoted in the 1990s (15). As a result, the annual production of bullfrogs reached 600,000 tons in 2021 (16). American bullfrog farming is intensive, with animal densities reaching 15 to 25 kg/m^2^ in China (17). Due to this high-density farming coupled with environmental changes (18, 19), a variety of pathogens have been reported to cause large-scale production losses of cultured bullfrogs, including Batrachochytrium dendrobatidis (20), ranavirus (21), Aeromonas hydrophila (22), Streptococcus iniae (23), Vibrio cholerae (24), and Elizabethkingia miricola (25). Bacterial pathogens in particular have become the focus of bullfrog breeding prevention and control. From September to October 2021, a severe infectious disease broke out at a bullfrog farm in Guangzhou, China. Infected bullfrogs weighed 40 to 60 g, and the total cumulative mortality on these farms ranged from 20% to 30%. Clinical signs in infected bullfrogs included congestive enlargement with inflammation in multiple organs, including the liver, spleen, kidneys, and lungs. In this study, we isolated, purified, and cultured the pathogenic strain from diseased organs and identified it as K. pneumoniae by analyzing its growth, biochemical, and phylogenetic characteristics. Its pathogenicity was confirmed by artificial infection experiments in bullfrogs and tadpoles.

## RESULTS

### Clinical signs of natural infection.

**FIG 1:**
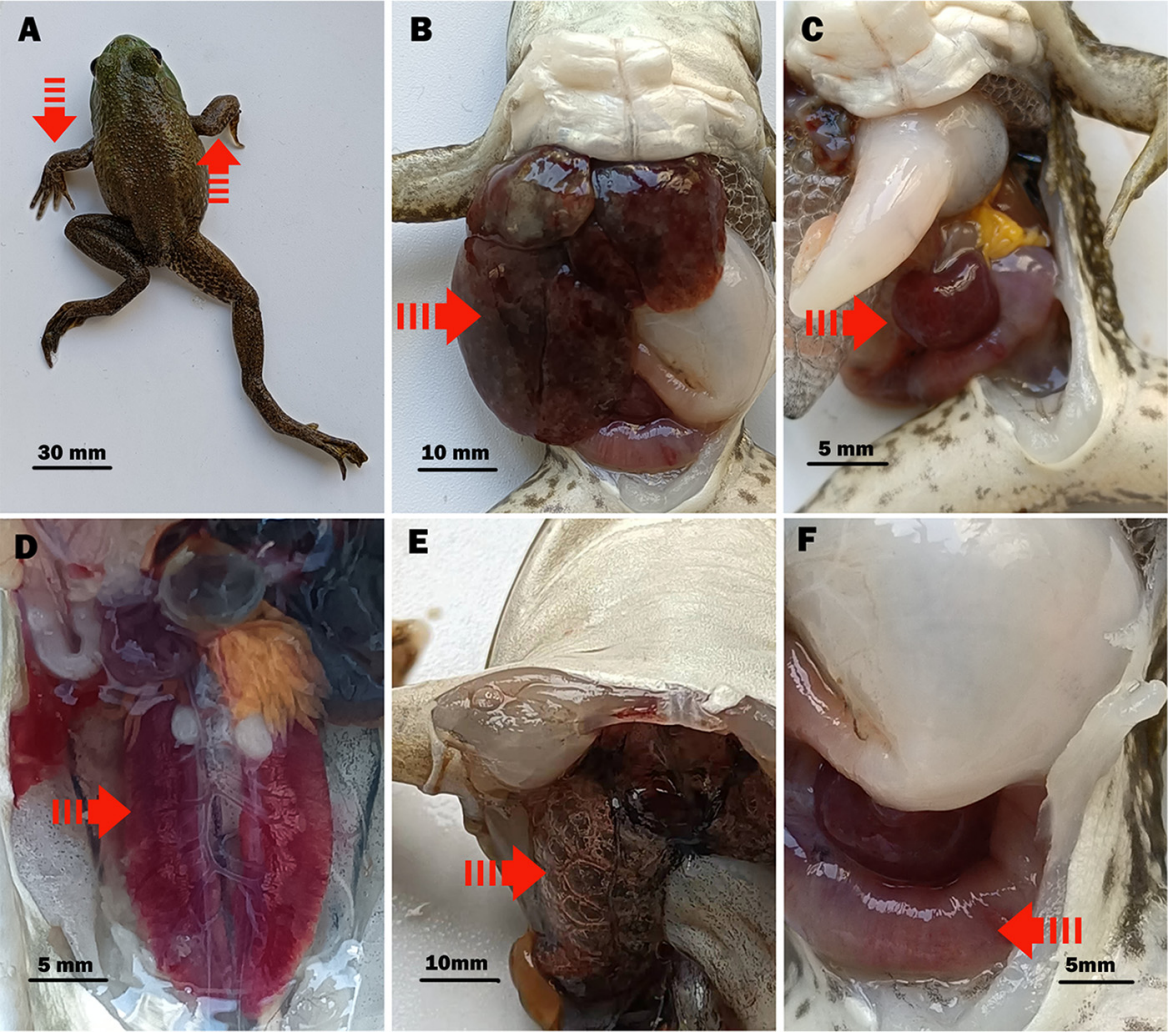
Clinical symptoms of bullfrog naturally infected with Klebsiella pneumoniae. (A) Symptoms, spasm symptoms (arrow). (B) Severe organ lesions, hepatic congestive enlargement, and hemorrhagic necrosis (arrow). (C) Severe organ lesions, congestive splenomegaly (arrow). (D) Renal congestive enlargement (arrow). (E) Congestive emphysema (arrow). (F) Congestive intestinal edema (arrow).

In infected bullfrogs, crawling weakness and spasms (Fig. 1A) were observed in 4 of 45 bullfrogs (9%). The dissected tissues showed hepatic congestive enlargement and extensive hemorrhagic necrosis (Fig. 1B) in 34 of 45 bullfrogs (76%). The spleen (Fig. 1C), kidneys (Fig. 1D), and lungs (Fig. 1E) were congested and enlarged, and 31 (69%), 31 (69%), and 29 bullfrogs (64%) showed these signs, respectively. In addition, congestive intestinal edema (Fig. 1F) was observed in 21 bullfrogs (47%).

### Morphologic and biochemical characteristics of the isolate strain.

**FIG 2:**
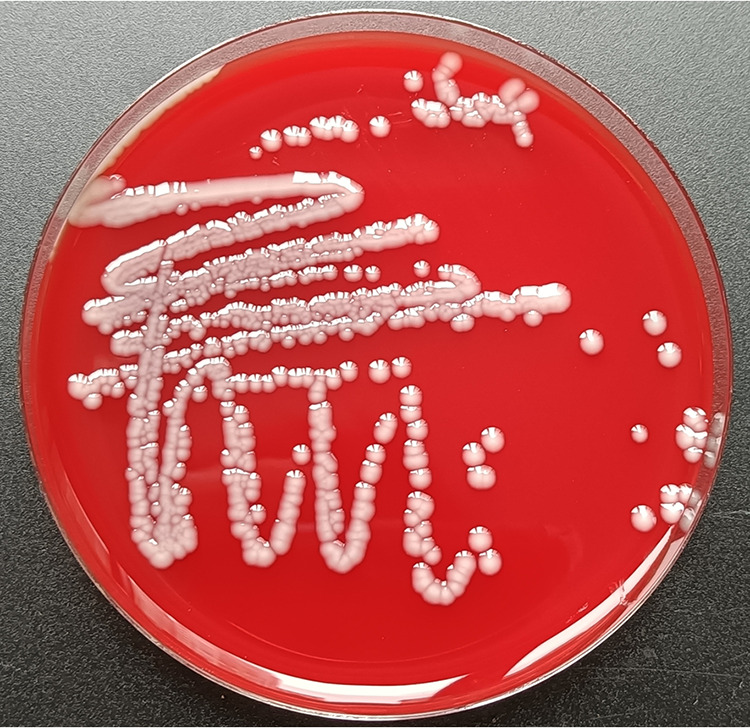
Colony morphology of K. pneumoniae NW202109.

**FIG 3:**
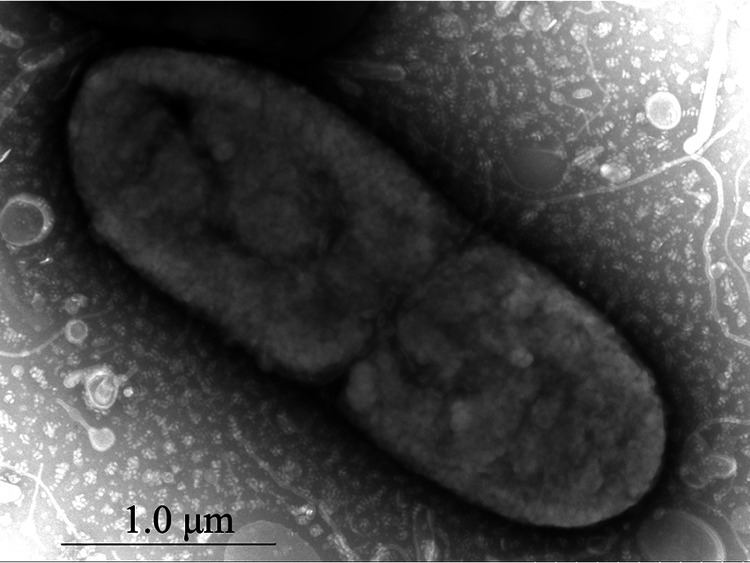
Transmission electron micrograph of the K. pneumoniae NW202109 strain.

Fifteen purified isolates were obtained from diseased bullfrogs. The 16S rRNA gene sequences of all isolates were amplified and sequenced. Sequencing analyses showed that the 16S rRNA genes of forty-five isolates were 99.9% to 100% homologous. One bacterial isolate was selected and named NW202109 for further identification and detailed investigation. The isolate NW202109 produced large, dome-shaped, sticky colonies with diameters of 0.8 to 2.2 mm on 5% sheep blood medium (Fig. 2). Transmission electron microscopy showed the strain is a short rod, 0.6 to 4 μm long and 0.3 to 1.5 μm wide (Fig. 3). The physiological and biochemical identification results were consistent with the characteristics of K. pneumoniae (Table S1).

### Phylogenetic analysis based on 16S rRNA, *rpoB*, and *gyrB* gene sequences.

**FIG 4:**
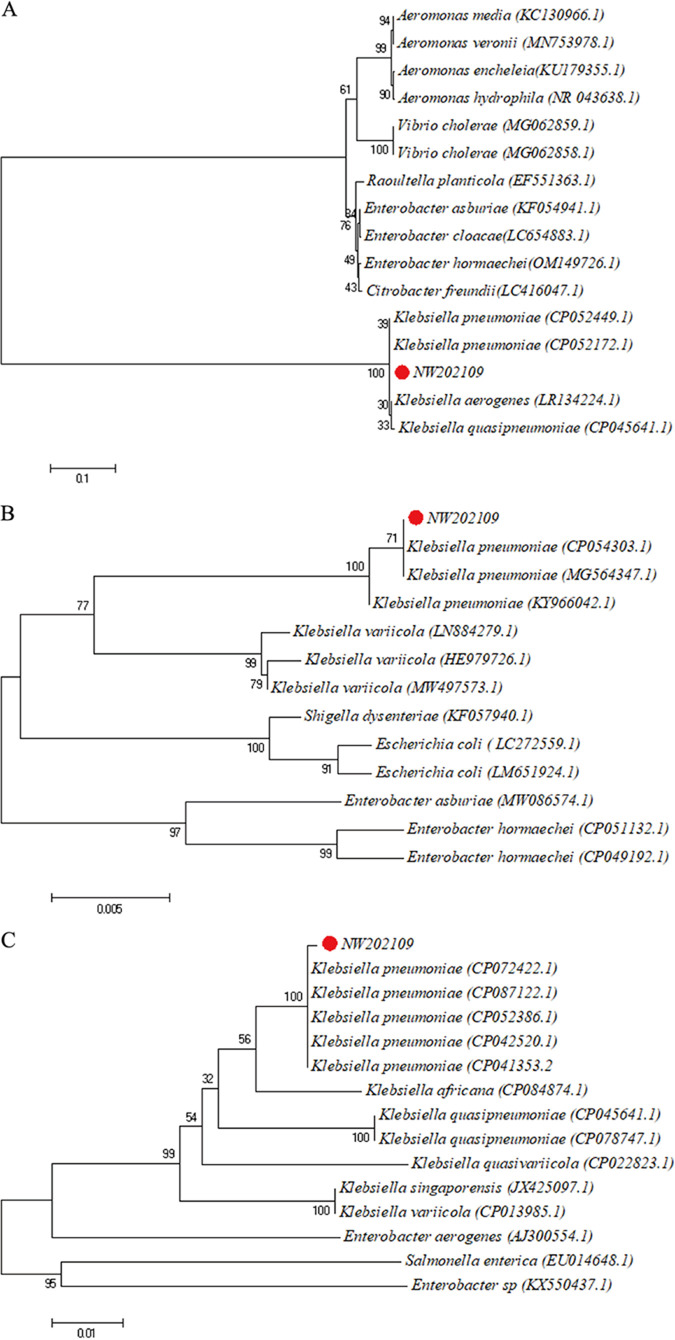
Phylogenetic trees constructed from (A) 16S rRNA gene sequences, (B) *rpoB* gene sequences, and (C) *gyrB* gene sequences of K. pneumoniae NW202109.

The 16S rRNA, *rpoB*, and *gyrB* partial gene sequences of NW202109 were determined and submitted to the GenBank database (accession no. OM530219, OM638428, OM638427, respectively). The sequences of 16S rRNA, *rpoB*, and *gyrB* were most similar to those of K. pneumoniae, with 97.8% (CP052449.1), 99.04% (CP054303.1), and 99.87% (CP072422.1) identity, respectively. Therefore, the phylogenetic trees were constructed based on the same gene sequences and a homologous series of NW202109, as shown in Fig. 4. In the phylogenetic tree derived from the 16S rRNA gene, NW202109 clustered with three species of Klebsiella. In the phylogenetic trees derived from the *rpoB* and *gyrB* gene sequences, NW202109 clustered with K. pneumoniae.

### Growth characteristics of NW 202109.

**FIG 5:**
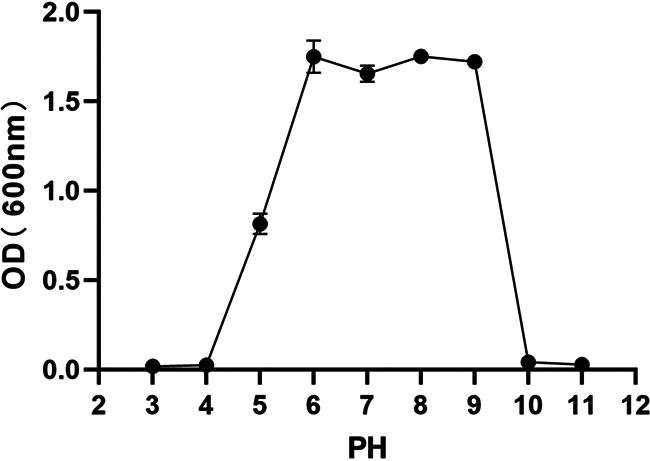
Effect of pH on K. pneumoniae NW202109 growth.

**FIG 6:**
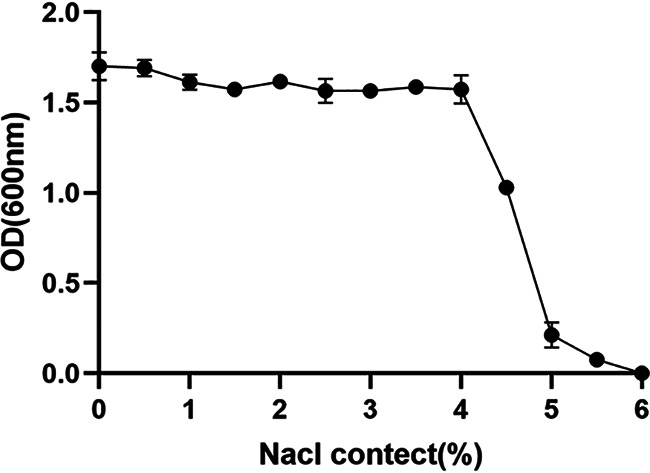
Effect of salinity on K. pneumoniae NW202109 growth.

NW 202109 survived at a pH of 3 to 11 and grew best at a pH of 6 to 9, with an optical density (OD) of 1.7 to 1.75 (Fig. 5). Limited growth was observed at pH 3, 4, 10, and 11. NW 202109 cultured well in media containing 0% to 4% Nacl (wt/vol). However, when the NaCl content reached 4.5%, bacterial growth was gradually inhibited; it was more strongly inhibited at 5% and significantly inhibited at a NaCl concentration of 5.5% (Fig. 6).

### The Klebsiella pneumoniae NW 202109 infection challenge experiment.

**FIG 7:**
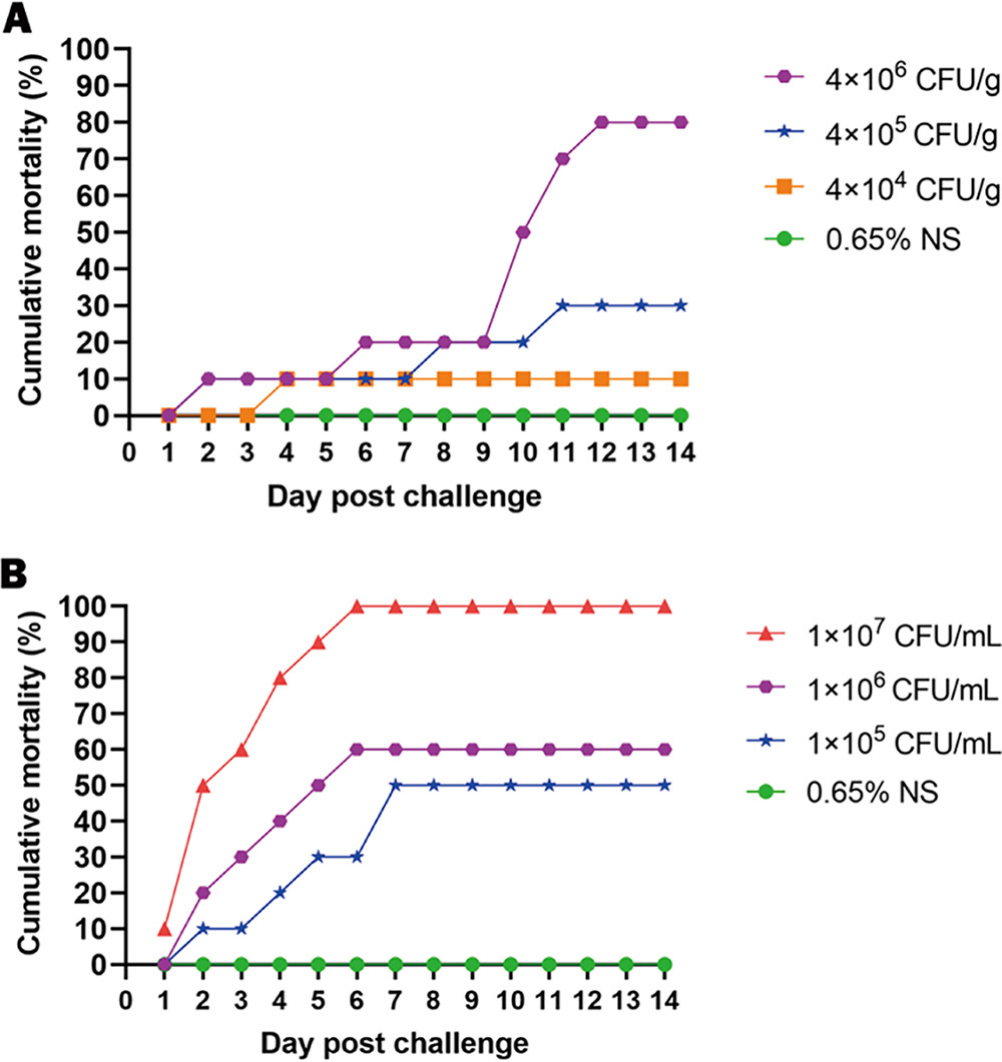
Cumulative daily mortality of (A) bullfrogs following intramuscular challenge experiments and (B) tadpoles following immersion challenge experiments with K. pneumoniae NW202109.

Bullfrogs with the highest bacterial challenge dose (4 × 10^6^ CFU/g) showed abdominal distention, slow movement, and one acute death 2 days after artificial infection. High mortality occurred between 10 and 12 days after infection, and the cumulative mortality rate reached 80%. When the bacterial challenge dose dropped to 4 × 10^5^ CFU/g, the bullfrogs showed similar signs, one acute death occurred, and the final cumulative mortality decreased to 30% (Fig. 7A). In contrast, only one of the bullfrogs infected with the lowest dose (4 × 10^4^ CFU/g) died on the fourth day, and no significant signs were observed in the remaining infected bullfrogs. No deaths or visible changes were observed in the control groups. Artificially infected bullfrogs were dissected and showed congestive enlargement of multiple organs, similar to that of the naturally infected bullfrogs. K. pneumoniae was isolated and identified from all dying infected bullfrogs.

**FIG 8:**
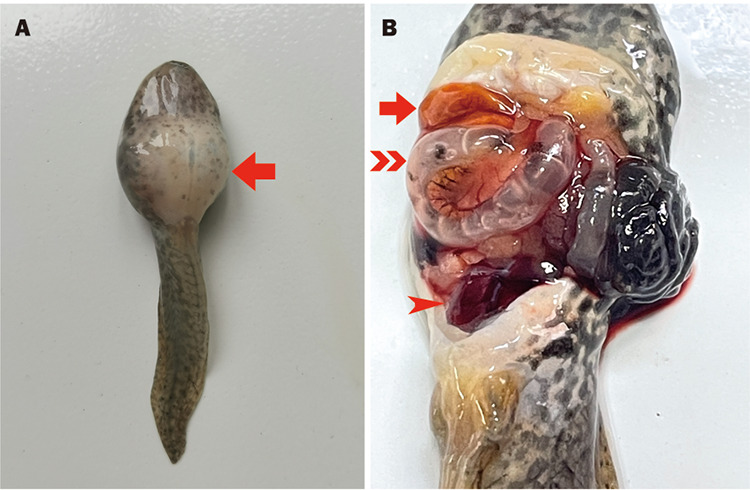
Tadpoles following K. pneumoniae NW202109 infection. (A) Superficial symptoms, abdominal enlargement (arrow). (B) Organ and tissue lesions: liver, congestive (arrow); kidney enlargement (arrowhead); and intestinal flatulence (double-headed arrow).

Tadpoles immersed in the highest infection concentration (1 × 10^7^ CFU/mL) began to bloat and swim in rolls, and one died on the first day after the immersion experiment (Fig. 7B). When the bacterial suspension concentration decreased to 1 × 10^6^ and 1 × 10^5^ CFU/mL, the infected tadpoles showed signs relatively later, and cumulative mortality decreased to 60% and 50%, respectively. No deaths or visible changes were observed in the control groups. Anatomical examination revealed congestive enlargement in the liver, kidneys, and intestines of the infected tadpoles, and the signs were similar to those in the infected bullfrogs (Fig. 8). K. pneumoniae was isolated and identified from all dying infected tadpoles.

### Histopathological analysis.

**FIG 9:**
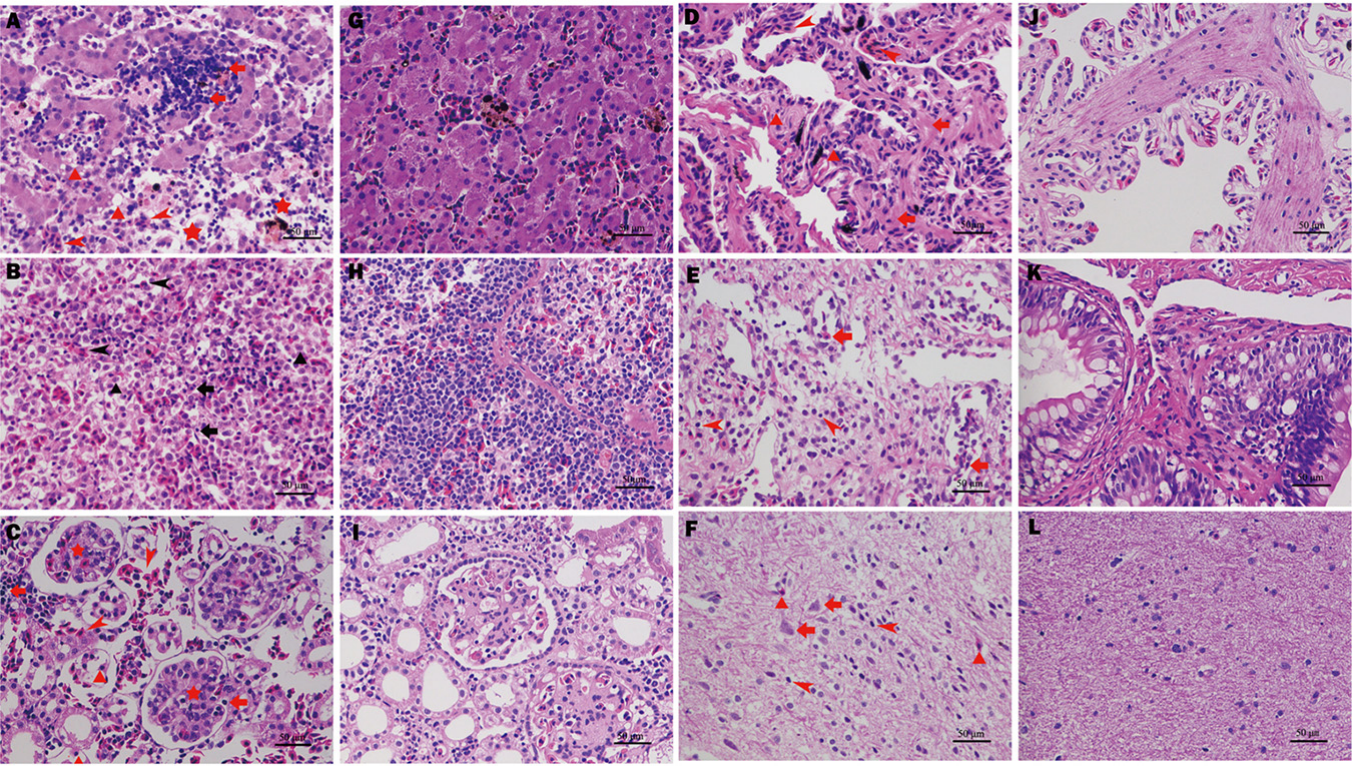
Histopathological changes in bullfrogs infected with K. pneumoniae NW202109. (A) Pathological changes in the liver: inflammatory reaction of liver tissue was severe. Many lymphocytes are seen between the hepatic plates, with a few plasma cells (arrow); hepatic sinusoid was hyperemic (arrowhead); hepatocellular vacuolar degeneration (triangle). Some hepatocytes were dissolved, and necrotic foci were observed (pentagram). (B) Pathological changes in the spleen: significant reduction of lymphoid tissue in the spleen (arrow); red pulp was markedly hyperplastic; sinus was dilated (triangle); hyperaemia (arrowhead). (C) Pathological changes in the kidney: infiltration of small clusters of inflammatory cells, predominantly lymphocytes and eosinophils (arrow); renal tubule epithelia and brush border were detached and degenerated (arrowhead); glomerular mesangial cell and mesangial matrix degeneration (pentagram); hyperemia, edema, and hemorrhage (triangle). (D) Pathological changes in the lungs: stromal fibrous proliferation (arrow) and hyperaemia (arrowhead); infiltration of lymphocytes and eosinophils (triangle). (E) Pathological changes in the intestinal tract: hyperemia, edema, hemorrhage (arrow); degeneration with various degrees of inflammatory reaction (arrowhead). (F) Pathological changes in the brain: neuronal degeneration (arrow); inflammatory cells, mainly lymphocytes (arrowhead); slight hyperaemia (triangle). (G to L) Negative control.

Similar pathological features were observed in all infected bullfrogs, characterized by hyperaemia and associated inflammation of multiple organs. Several animals showed slight congestion in the brain. The inflammatory reaction of the liver was severe. Many lymphocytes were seen between the hepatic plates with some plasma cells. The hepatic sinusoid was hyperemic. Some hepatocytes dissolved, and necrotic foci were observed. Hepatocellular vacuolar degeneration was found (Fig. 9A). The splenic tissue showed loss of typical architecture, such as loss of the normal ratio of white pulp to red pulp. The lymphoid tissue was markedly reduced, and no intact lymphoid follicles or periarteriolar lymphoid sheath were seen. The red pulp was markedly hyperplastic. The sinus was dilated and congested (Fig. 9B). Changes to the kidney mainly consisted of hyperaemia and inflammatory cell infiltration. Interstitial inflammation was evident, with small clusters of inflammatory cells, predominantly lymphocytes. Glomerular mesangial cells and the mesangial matrix were degenerated and hyperemic, with a small number of inflammatory cells, mainly lymphocytes (Fig. 9C). The alveolar walls were markedly thickened and hyperemic. Stromal fibrous proliferations infiltrated by lymphocytes and eosinophils were observed (Fig. 9D). The main pathological features in intestinal tissue were hyperemia, edema, hemorrhage, and degeneration, with various degrees of inflammatory reaction in the mucosal and submucosal layers (Fig. 9E). The brain tissue was slightly disorganized with inflammatory cell infiltration in the neuropil, predominantly lymphocytes. Neurons were mildly degenerated and exhibited giant-cell change. In addition, the brain tissue was slightly hyperemic (Fig. 9F).

**FIG 10:**
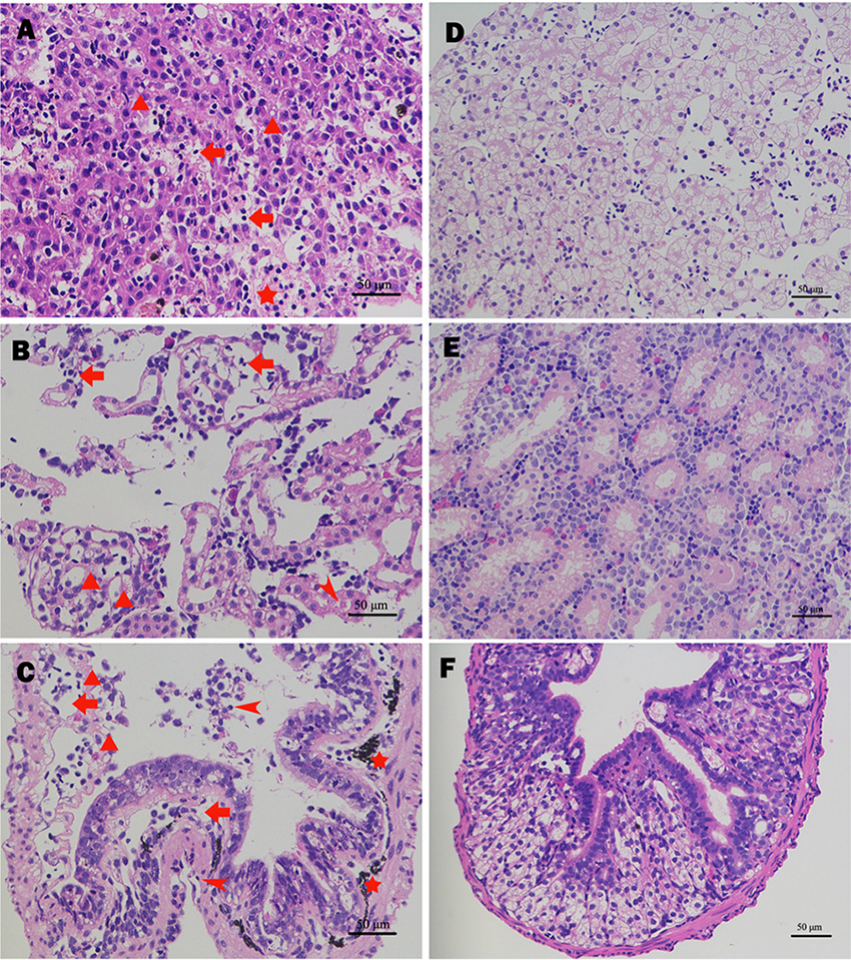
Histopathological changes in tadpoles infected with K. pneumoniae NW202109. (A) Pathological changes in the liver: inflammation of the liver tissue was evident, with small clusters of lymphocytes infiltrating between the hepatic plates (arrow); hepatocellular steatosis (triangle); focal hepatocyte necrosis (pentagram). (B) Pathological changes in the kidney: inflammatory cell (arrow); renal tubule epithelia and brush borders were detached and degenerated (arrowhead); glomerular mesangial cell and mesangial matrix degeneration (triangle). (C) Pathological changes in the intestinal tract: hyperemia, edema, hemorrhage (arrow); inflammatory cell (arrowhead); corrosive focus and ulcer (triangle) and pigmentation (pentagram). (D to F) Negative control.

The pathological changes in tadpoles were similar to those in bullfrogs. Inflammation of the liver tissue was evident with small clusters of lymphocytes between the hepatic plates. Severe steatosis and focal hepatocyte necrosis were found in liver cells (Fig. 10A). Changes to the kidney mainly consisted of hyperaemia and inflammatory cell infiltration. Interstitial inflammation was obvious, with small clusters of inflammatory cells, predominantly lymphocytes. Glomerular mesangial cells and the mesangial matrix were degenerated and hyperemic, with a small number of inflammatory cells, mainly lymphocytes (Fig. 10B). The main pathological features of the intestinal tissues were hyperemia, edema, hemorrhage, corrosive focus, and ulcers, with various degrees of inflammatory reaction in the mucosal and submucosal layers (Fig. 10C).

### Antibiotic susceptibility analysis.

Following antibiotic susceptibility testing, NW202109 was shown to be sensitive to gentamicin, terramycin, norfloxacin, ciprofloxacin, sulfamethizole, sulfapyridine, and sulfamethoxazole-trimethoprin (SMZ-TMP); intermediately susceptible to aztreonam and doxycycline; and resistant to penicillin, ampicillin, imipenem, neomycin, erythrocin, medemycin, and vancomycin. These results were the same at both 35°C and 28°C. In addition, NW202109 was sensitive to kanamycin at 35°C and intermediate at 28°C, and sensitive to chloramphenicol at 35°C and resistant at 28°C (Table 1).

**TABLE 1:** Susceptibility of NW202109 to antibiotics^*a*^

Antibiotic	Judgment standard of inhibition zone diam (mm)			Content (μg/disk)	Inhibition zone diam (mm)	
	Resistant	Intermediately sensitive	Sensitive		35°C	28°C
β-Lactams						
Penicillin	≤17	18–20	≥21	10	0^*b*^	0^*b*^
Ampicillin	≤13	14−16	≥17	10	0^*b*^	0^*b*^
Imipenem	≤13	14−15	≥16	10	10.41 ± 0.17^*b*^	11.92 ± 0.95^*b*^
Aminoglycosides						
Kanamycin	≤13	14−17	≥18	30	19.36 ± 0.34^*c*^	15.97 ± 0.54^*d*^
Gentamicin	≤12	13−14	≥15	10	17.67 ± 0.40^*c*^	20.85 ± 0.07^*c*^
Neomycin	≤12	13−16	≥17	30	0^*b*^	0^*b*^
Macrolides						
Erythrocin	≤13	14−22	≥23	15	0^*b*^	0^*b*^
Medemycin	≤13	14−17	≥18	30	0^*b*^	0^*b*^
Aztreonam	≤15	16−21	≥22	30	20.63 ± 0.53^*d*^	19.16 ± 0.67^*d*^
Tetracyclines						
Terramycin	≤14	15−18	≥19	30	20.31 ± 0.15^*c*^	19.82 ± 0.63^*c*^
Doxycycline	≤12	13-15	≥16	30	15.42 ± 0.39^*d*^	14.27 ± 0.30^*d*^
Quinolones						
Norfloxacin	≤12	13-16	≥17	10	24.42 ± 0.31^*c*^	26.35 ± 0.15^*c*^
Ciprofloxacin	≤15	16−20	≥21	5	23.91 ± 0.30^*c*^	28.01 ± 0.33^*c*^
Amphenicols						
Chloramphenicol	≤12	13−17	≥18	30	19.99 ± 0.66^*c*^	9.64 ± 0.29^*b*^
Sulfonamides						
Sulfamethizole	≤12	13−16	≥17	100	21.93 ± 0.33^*c*^	22.60 ± 1.46^*c*^
Sulfapyridine	≤12	13−16	≥17	300	21.64 ± 0.32^*c*^	22.12 ± 0.42^*c*^
SMZ-TMP	≤10	11−15	≥16	25	18.33 ± 0.28^*c*^	24.43 ± 0.14^*c*^
Glycopeptides						
Vancomycin	≤14	15−16	≥17	30	0^*b*^	0^*b*^

a SMZ-TMP, sulfamethoxazole and trimethoprin combination.

b Resistant.

c Sensitive.

d Intermediately sensitive.

## DISCUSSION

In this study, a pathogenic strain of K. pneumoniae (NW202109) was isolated from diseased American bullfrogs. Infection experiments showed that the strain was highly virulent to bullfrogs, and the clinical signs in infected bullfrogs were similar to those observed in naturally diseased bullfrogs. The same pathogenic bacteria were then isolated from the tissues of experimentally infected bullfrogs, fulfilling Koch’s postulates (26). The results of bacterial identification showed that NW202109 was a strain of K. pneumoniae. K. pneumoniae is a well-known primary pathogen of terrestrial organisms and, in recent years, has been considered an opportunistic bacterial pathogen of a variety of aquatic animals. To our knowledge, this was the first report of Klebsiella pneumoniae infecting American bullfrogs or, indeed, any amphibians.

The accurate identification of bacterial isolates is crucial for diagnosis and effective treatment of infectious diseases. In the 1980s, it was discovered that some bacterial species lack distinct physiological and biochemical indicators (27). However, modern advancements in automatic identification instruments are considered reliable methods for characterization (28) and could be used to supplement molecular analyses for bacterial identification. Moreover, housekeeping genes have been proven to be effective for the molecular identification of bacterial species. This study used three housekeeping genes (16S rRNA, *rpoB*, and *gyrB)* for bacterial identification. Sequence analysis based on the 16S rRNA gene is a standard method of bacterial identification and was once considered the “gold standard” for bacterial identification (29). The RNA polymerase β subgene unit *ropB* is involved in DNA transcription and can also be used as a target gene for species identification (30). The *gyrB* gene is involved in DNA replication and repair and has a high resolution in species identification (31). *gyrB* is considered more reliable than phylogenetic trees based on 16S rRNA for identifying K. pneumoniae from both animal and plant sources (32, 33). In this study, we used the BD Phoenix Automated Microbiology System for the identification of isolated strains: the Phoenix system is a proven and reliable method for identifying the majority of clinical strains encountered (34). Combined with physiological and biochemical characteristics and the homology of three housekeeping genes, the pathogenic bacterium was identified as K. pneumoniae.

Interestingly, the analysis of growth characteristics showed that NW202109 could live in a wide salinity range. The growth of many terrestrial bacteria is completely inhibited at a salinity of 2% to 3%, and many aquatic bacteria cannot survive at a salinity of 4% to 4.5% (35–37), but NW202109 could grow slowly at a salinity of 5.5%. K. pneumoniae has been confirmed to possess three electrogenic Na^+^/H^+^ exchangers (38), which may be the key to its adaptation to different salinities.

Infection challenges evaluated the virulence of the strain in healthy bullfrogs. The results showed that the strain could cause pathological changes and even death in bullfrogs. After the onset of symptoms, acute death of bullfrogs was rare, and the diseased organs of individual bullfrogs were not always consistent, with lesions in all organs or in only parts of organs. With the progression of organ disease, differentiation of death and signs relief occured. Moreover, the disease progression was dose-dependent. In this study, the typical signs of K. pneumoniae NW 202109 infection in bullfrogs were similar to those reported in other animals, including congestive edema, necrosis, and inflammation in multiple organs (11, 39). These pathological characteristics were partly consistent with the pathogenic characteristics of hypervirulent K. pneumoniae (40) and can also cause a variety of human diseases (2). K. pneumoniae can evade and actively inhibit the innate immune responses of a host (41) and is not a species-specific pathogen (42), which may account for its widespread infection of animals.

Damage to multiple organs and congestive edema in bullfrogs infected with K. pneumoniae are similar symptoms to those of bullfrogs infected with *E. miricola* (25) and A. hydrophila (22). Therefore, misdiagnosis of the infection due to anatomical analyses could occur. However, K. pneumoniae-infected bullfrogs did not show signs of torticollis and rotational swimming, as in those infected with *E. miricola*. Moreover, the animals did not show “red legs” symptoms like those in A. hydrophila-infected animals. Surprisingly, some infected bullfrogs also exhibited convulsive signs. K. pneumoniae was isolated from the brains of infected bullfrogs. Microscopic examinations revealed noticeable pathological changes in the brain. Only *E. miricola* had previously been identified to infect the bullfrog brain, with different symptoms from those caused by K. pneumoniae, which should be further studied.

Tadpoles were also used in challenge experiments. The results showed that the apparent symptoms and pathological features in infected tadpoles were similar to those in bullfrogs, including abdominal distension, multiple organ enlargement, and bleeding. In addition, the infected tadpoles rapidly developed symptoms such as side-swimming. K. pneumoniae was also isolated from the brain. Hatchling health is crucial in aquaculture, and our experimental results indicate that the long-term survival of K. pneumoniae in tadpoles will significantly impact bullfrog culture. Therefore, it is essential to pay attention to prevention and control of K. pneumoniae during the hatchling stage in bullfrog aquaculture.

Drug-resistant K. pneumoniae is considered one of the most urgent public threats worldwide (43), and antimicrobial resistance in the NW202109 strain was identified in this study. Our data showed that this bacterium was resistant to glycopeptides, macrolides, β-lactams, carbapenems, and neomycin and had intermediate resistance to doxycycline and aztreonam according to CLSI criteria. This suggested that NW202109 was a typical multidrug-resistant K. pneumoniae strain. Drug-resistance genes can be transferred to new strains of the same or other bacterial genera by plasmids, phages, and other genetic elements (44, 45). Therefore, the spread of multidrug-resistant clones of K. pneumoniae is of great concern (46). In addition, our experiments demonstrated an interesting result: NW202109 was sensitive to both kanamycin and chloramphenicol at 35°C, but intermediately susceptible to kanamycin, and resistant to chloramphenicol at 28°C (the optimum growth temperature for bullfrog cells). Membrane damage caused by temperature changes would impact bacterial susceptibility to a variety of antimicrobials through their initiation of stress responses (47), increasing the difficulty of medication. As a result, K. pneumoniae has been included in the WHO priority list of pathogens which urgently need new drugs (48), and it is important to identify and screen sensitive drugs to control these strains. We have shown that NW202109 is sensitive to two quinolones and three sulfonamides, providing reference drugs for disease control in bullfrog farming.

## MATERIALS AND METHODS

### Clinical examination.

Forty-five infected bullfrogs, weighing approximately 40 to 60 g, were obtained from a bullfrog farm in Guangzhou, China. Clinical signs were observed, and internal organs were dissected and analyzed.

### Experimental animals.

Healthy bullfrogs weighing 50 ± 5 g and healthy tadpoles weighing 5 ± 0.5 g were acquired from the same bullfrog farm in Guangzhou, China. The bullfrogs and tadpoles were maintained in 500-L aquaculture tanks under laboratory conditions at 25 ± 2°C for 2 weeks before the experiments were conducted. The water was changed daily. All bullfrogs used in the experiments were anesthetized with eugenol, and all protocols for experiments involving live animals conducted in this study were approved by the Animal Care and Use Committee of the Zhongkai University of Agriculture and Engineering (approval no. ZK 20210920001).

### Bacterial isolation.

Fifteen moribund bullfrogs from affected farms were dissected using aseptic techniques. Tissue samples were taken from the liver, kidney, and spleen. The tissues were used to inoculate a 5% sheep blood medium (Detgerm, Guangzhou, China) to culture the bacterium for 18 h at 28°C for isolation and purification. The resulting single colonies were subcultured on the same medium to check the purity of the isolated strain.

### Biochemical characteristics of isolate strain.

Before the experiment, the isolates were subcultured overnight in brain-heart infusion (BHI) broth (Huankai, Guangzhou, China) at 28°C. The biochemical characteristics, hydrolytic activity, and carbon source utilization of isolated bacteria were evaluated using the BD Phoenix Automated Microbiology System (BD Biosciences, Franklin Lakes, NJ, USA). Morphology was characterized using a HT7800 transmission electron microscope (Hitachi, Santa Clara, CAUSA).

### Growth characteristics of isolated strain.

In this study, we verified the effects of pH and salinity on the survival and growth of the bacterium. The sterile BHI culturing broths varied from pH 3 to 11. At each pH gradient, 5 mL of sterile BHI broth was inoculated with 50 μL of bacterial suspension (2 × 10^7^ CFU/mL) and incubated at 28°C. The salinity of sterile BHI broth was adjusted to 0% to 6% over 13 gradients. For salinity, 5 mL sterile BHI broth was inoculated with 50 μL of bacterial suspension (2 × 10^7^ CFU/mL) and incubated at 28°C for each gradient. All samples were cultured at 200 rpm/min for 18 h, and the concentration of the culture was detected by measuring the absorbance of the culture at 600 nm (*A*_600_).

### Phylogenetic analysis.

DNA from the isolated bacterium was extracted with a genomic DNA extraction kit (Vazyme, Nanjing, China) following the manufacturer's guidelines. The DNA was stored at −80°C until required for genetic analysis. PCR amplification primers for the 16S rRNA, *gyrB*, and *rpoB* genes were used to identify the specific bacterial strain as described by Liu et al. (35). The PCRs were conducted in a 50-μL volume with 25 μL 2× *Taq* HiFi PCR mix (Mei5 Biotech, Beijing, China), 21.5 μL sterile double-distilled water, 1 μL DNA template (100 ng), and 1.25 μL of each specific primer (10 mm).Thermal cycler conditions were set as follows: 94°C for 5 min; 32 cycles of 95°C for 25 s, 56°C for 25 s, 72°C for 30 s, and 72°C for 5 min; and storage at 4°C. The PCR products were sequenced directly by Tianyi Huiyuan Gene Technology Co., Ltd., with sample preparation following the manufacturer’s instructions. Sequence analysis was performed using the BLAST database network service and MEGA v7.0.20. The adjacency neighbor-joining tree algorithm, Kimura 2-parameters, and 1,000 bootstrap copies were used to infer the unrooted evolutionary tree.

### Challenge experiment.

To determine the pathogenicity of the isolated bacterium, challenge experiments were conducted using healthy bullfrogs and tadpoles. Both bullfrogs and tadpoles were split into four groups: three groups were challenged, and one served as a negative control.

Ten bullfrogs were each challenged with 200 μL of the bacterium suspended in 0.65% sterile normal saline (NS) through intramuscular injection. For each bullfrog group, the concentration of the bacterial solution was adjusted to 1 × 10^9^, 1 × 10^8^, and 1 × 10^7^ CFU/mL, respectively; the negative-control group received a 0.65% NS injection. For the tadpole groups, 10 tadpoles from each group were immersed in 2 L bacterial suspension for 20 min at concentrations of 1 × 10^7^, 1 × 10^6^, and 1 × 10^5^ CFU/mL, respectively. Tadpoles in the control group were immersed in 0.65% NS water. After the immersion challenge, all the tadpoles were maintained in aerated freshwater. Clinical signs and mortality were observed daily for 14 days, and bacteria were isolated and identified from infected bullfrogs and tadpoles.

### Histopathological analysis.

The brain, heart, liver, spleen, kidney, intestine, and lung of the infected bullfrogs with obvious signs were fixed with 4% (wt/vol) paraformaldehyde for 96 h, dehydrated with 80%, 90%, 95%, and 100% graded concentrated ethanol, made transparent with xylene, and embedded in paraffin, and sections were made from 3 to 5 μm. Sections were stained by Mayer’s Hematoxylin and Eosin and observed by light microscopy.

### Antimicrobial susceptibility testing.

The isolated strain was screened for sensitivity to 18 antibiotics using the Kirby Bauer disk-diffusion method (49). For the suspension, 100 μL of the bacterium at a concentration of 1 × 10^9^ CFU/mL was tiled onto Mueller-Hinton agar plates prepared with drug-sensitive tablets (Binhe, Hangzhou, China). The inoculated plates were cultured at 35°C and 28°C for 24 h to determine the inhibition zone diameter. According to the M100 Performance Standards for Antimicrobial Susceptibility Testing, 31st edition (50), the results were labeled “sensitive,” “intermediately sensitive,” and “drug-resistant.”
