# Supplementary material for: Identification and Characterization of Klebsiella pneumoniae from Farmed American Bullfrogs (Rana catesbeiana)
Source: Microbiol Spectr. 2023 Jan 5;11(1):e03579-22. doi: 10.1128/spectrum.03579-22 (PMC9927386; doi:10.1128/spectrum.03579-22)
Supplement: Supplemental file 1 — Table S1. Download spectrum.03579-22-s0001.pdf, PDF file, 0.1 MB [file spectrum.03579-22-s0001.pdf]

TABLE S1 Biochemical and physiological characteristics of the isolated strain NW202109

Identified items	Results	expected value
Acetic acid	-	V
citric acid	+	+
a-ketoglutaric acid	+	+
Dextrose	+	+
B-gentiobiose	+	+
D-galactose	+	+
Sorbitol	+	+
D-Fructose	+	+
Galacturonic acid	+	+
Maltose V. P	+	+
Arg-Arg-7-amino-4-methylcoumarin	+	V
L-Leucine-7-amido-4-methylcoumarin	+	V
Glutaryl-Gly-Arg-7-amido-4-methylcoumarin	-	-
colimycin	+	-
Malonic ester	-	V
4-Methylumbelliferyl-N-Acetylβ-D-glucopyranoside	-	-
P-nitrophenol β- D-glycoside	+	+
Gly-Pro-7-amido-4-methylcoumarin	-	-
L-Arg-7-amido-4-methylcoumarin	-	-
L-Phe-7-amido-4-methylcoumarin	-	-
L-Trp-7-amido-4-methylcoumarin	-	-
Ribitol	+	+
D-Mannitol	+	+
L-Sodium Proline	-	-
D-gluconic acid	+	+
Sucrose	+	+
L-Rhamnose	+	+

L-Arabinose	+	+
Urea	-	V
Ornithine	-	-
Esculin	+	+
(p-nitrophenol) phosphoric acid Bis	+	V
N-acetyl-galactosamine	-	V
Gly-7-amido-4-methylcoumarin	-	-
L-Glutamic acid-7-amido-4-methylcoumarin	+	V
L-Pro-7-amido-4-methylcoumarin	-	-
Lys-Ala-7-amido-4-methylcoumarin -	-	-
crotonic acid	-	-
β -allose	-	-
D-Melibiose	+	+
Methyl β- Glycosides	+	+
N-acetyl-glucosamine	+	+
Y-L-Glutamine sodium	+	V

+:Positive；-:Negative；V：Variant in different stains
